# Genome-Wide Analysis of the Lysine Biosynthesis Pathway Network during Maize Seed Development

**DOI:** 10.1371/journal.pone.0148287

**Published:** 2016-02-01

**Authors:** Yuwei Liu, Shaojun Xie, Jingjuan Yu

**Affiliations:** State Key Laboratory for Agrobiotechnology, College of Biological Sciences, China Agricultural University, No. 2 Yuanmingyuan West Road, Beijing, 100193, China; La Trobe University, AUSTRALIA

## Abstract

Lysine is one of the most limiting essential amino acids for humans and livestock. The nutritional value of maize (*Zea mays* L.) is reduced by its poor lysine content. To better understand the lysine biosynthesis pathway in maize seed, we conducted a genome-wide analysis of the genes involved in lysine biosynthesis. We identified lysine biosynthesis pathway genes (LBPGs) and investigated whether a diaminopimelate pathway variant exists in maize. We analyzed two genes encoding the key enzyme dihydrodipicolinate synthase, and determined that they contribute differently to lysine synthesis during maize seed development. A coexpression network of LBPGs was constructed using RNA-sequencing data from 21 developmental stages of B73 maize seed. We found a large set of genes encoding ribosomal proteins, elongation factors and zein proteins that were coexpressed with LBPGs. The coexpressed genes were enriched in cellular metabolism terms and protein related terms. A phylogenetic analysis of the LBPGs from different plant species revealed different relationships. Additionally, six transcription factor (TF) families containing 13 TFs were identified as the Hub TFs of the LBPGs modules. Several expression quantitative trait loci of LBPGs were also identified. Our results should help to elucidate the lysine biosynthesis pathway network in maize seed.

## Introduction

Lysine cannot be synthesized in the body and must be obtained from food, and consequently it is one of the most limiting essential amino acids for human and livestock. Lysine is highly limited in cereal grains [1].The poor lysine content of maize (*Zea mays* L.), one of the most important cereal crops, thus greatly reduces its nutritional value to monogastric animals [2].

Two different lysine biosynthesis pathways occur in nature. One is via α-aminoadipate which exists in fungi and *Euglena* [3]. The other is the diaminopimelate pathway which exists in bacteria, plants, and archaea [4]. Diaminopimelate pathway belongs to the aspartate family pathway in which aspartate kinase (AK) is the first enzyme that catalyzes aspartate phosphorylation to form aspartate-β-semialdehydea [5]. Dihydrodipicolinate synthase (DHDPS) catalyzes the first reaction unique to lysine biosynthesis, the condensation of aspartic-semialdehyde with pyruvate, to form dihydrodipicolinate. The reaction catalyzed by DHDPS is the primary regulation point in the diaminopimelate pathway because the activity of DHDPS is strictly feedback inhibited by lysine [6]. Tetrahydrodipicolinate (THDPA) is then produced from dihydrodipicolinate through the action of dihydrodipicolinate reductase (DapB). There are three variant pathways for converting THDPA to meso-diaminopimelate (m-DAP), which is the substrate of m-DAP decarbosxylase (LysA). One pathway is only catalyzed by m-DAP dehydrogenase (Ddh) [7]. The second one is the succinyl-dependent pathway in which LL-diaminopimelate (LL-DAP) is produced from THDPA through the action of tetrahydrodipicolinate succinylase (DapD), succinydiaminopimelate aminotransferase (DapC), and desuccinylase (DapE), and then converted to m-DAP catalyzed by diaminopimelate epimerase (DapF) [8]. The third one is similar to the second one except that the intermediates are acetylated instead of succinylated [9]. Recently, a fourth variant pathway was reported in Arabidopsis, in which THDPA is directly convert into LL-DAP through the action of LL-diaminopimelate aminotransferase (LL-ADP-AT), bypassing the DapD-, DapC- and DapE-catalyzed steps found in the second and third ones [10]. Alterations in the AK and DHDPS activities are used to increase the free lysine content and to improve cereal protein quality. Many recent studies involving the elevation of the free lysine content via the expression of bacterial feedback-insensitive AK and DHDPS have been conducted in plants, such as tobacco [11], canola, soybean [12], potato [13], and barley [14]. Monsanto has developed a high lysine maize cultivar, LY038, through the specific expression of a lysine feedback-insensitive DHDPS in the endosperm [15]. Additionally, Huang et al. also produced a high lysine maize line through genetic crosses between the feedback-insensitive DHDPS lines and the zein reduction lines [16].

The gene and pathway annotation databases [17,18] and next-generation deep-sequencing and RNA sequencing (RNA-Seq) technologies [19,20] have facilitated the genome-wide analysis of metabolic pathways. RNA-Seq can precisely measure the expression levels of transcripts and their isoforms [21]. Additionally, rapid advances in network biology have provided the opportunity to understand molecular interactions, including protein–protein interaction, metabolic signaling and transcriptional regulatory networks [22]. The weighted gene co-expression network analysis (WGCNA), a systems biology method for describing correlations among quantitative data sets, such as those obtained by microarray analyses and RNA-Seq, can be used to find modules of highly correlated genes [23]. WGCNA has been successfully used to study data from humans, mice, and plants [24–29]. Expression quantitative trait loci (eQTLs) mapping is a powerful tool by which gene regulatory networks are revealed using gene expression levels as quantitative traits. With the development of microarrays and RNA-Seq technologies, eQTL mapping has progressed greatly. eQTL mapping was first carried out in yeast [30], followed by mapping in human, mice, maize and Arabidopsis [31–36]. Based on physical distances from the regulated gene, eQTLs are split into two groups: local eQTLs (cis-eQTLs) are mapped near the target gene, which they may strongly influence, whereas distant eQTLs (trans-eQTLs) have subtle effects on the target gene [37].

In this study, using the Kyoto Encyclopedia of Genes and Genomes (KEGG) and Phytozome databases, we identified LBPGs and analyzed whether a diaminopimelate pathway variant exists in maize. We also explored the LBPGs phylogenetic relationships. Additionally, to reveal the lysine biosynthesis network during maize seed development, we constructed a coexpression network of LBPGs using publicly available RNA-Seq data from 21 developmental stages of B73 maize seed. We also analyzed differences between two DHDPS encoding genes, DHDPS1 and DHDPS2, and their coexpressed genes, and found that DHDPS1 and DHDPS2 play different roles in lysine biosynthesis in maize seed. Furthermore, eQTL mapping was used to identify genetic variants that regulated LBPG expression. Our results should increase our knowledge of the lysine biosynthesis network in maize seed.

## Materials and Methods

### Identification and phylogenetic analyses of LBPGs

The amino acid sequences of candidate LBPGs for maize and other species were obtained from the lysine biosynthesis pathway annotation of KEGG PATHWAY (http://www.kegg.jp/kegg-bin/show_pathway?org_name=map&mapno=00300&mapscale=1.0&show_description=hide). The LBPGs were selected based on the functional annotations and Enzyme Commission (EC) annotations in Phytozome 9.0 (http://www.phytozome.net) or when the KEGG amino acid sequence had 100% identity to the sequence in Phytozome. LBPG gene IDs were obtained by using the BLASTP algorithm to search Phytozome 9.0 with the corresponding genomes of each species. For each lysine biosynthesis enzyme, the phylogenetic trees were constructed with the full-length protein sequences of *Zea may* (v5b), *Sorghum bicolor* (v2.0), *Setaria italica* (v2.0), *Oryza sativa* (v7.0), *Brachypodium distachyon* (v2.0), *Arabidopsis thaliana* (TAIR10), *Medicago truncatula* (Mt4.0v1), *Populus trichocarpa* (v3.0), *Solanum lycopersicum* (v2.3), *Selaginella moellendorffii* (v1.0), *Physcomitrella patens* (v3.0) and *Chlamydomonas reinhardtii* (v5.0) using MEGA5 [38] by the neighbor-joining method with 500 bootstrap replicates.

### LBPG coexpression network construction

The WGCNA program in the R software package [25] was used, along with the step-by-step network construction and module detection method, to construct the coexpression network. The data used for the network were based on RNA-Seq data published by Chen [39] from 21 seed developmental stages of maize cultivar B73. Only genes expressed during at least two stages with the reads per kilobase per million reads (RPKM) values ≥ 2 or expressed at only one stage with an RPKM ≥ 5 were used for network construction. The weighted gene coexpression network during maize seed development was constructed using the Log_2_+1 normalized RPKM expression values of selected genes. The soft thresholding power β was used to calculate adjacencies. To minimize the effects of noise and false associations, we transformed the adjacencies into a topological overlap matrix (TOM), and calculated the corresponding dissimilarities, dissTOM, as 1 –TOM. To hierarchically cluster the genes, we used dissTOM as a distance measure and set the minimum module size (number of genes) to 100 to detect modules. To quantify the coexpression similarities of entire modules, the eigengenes of modules were calculated and clustered based on their correlation [40]. We choose the correlation of 0.9 to cluster the modules. The modules’ eigengenes were used to represent the gene expression pattern within a module [41]. Cytoscape (v 3.0.2) [42] was used to display the coexpression network.

### Annotation of coexpressed genes

The online tool WEGO (http://wego.genomics.org.cn) was used for the Gene Ontology (GO) enrichment analysis [43]. GO terms of genes (RefGen_v2 5b) were obtained from the maize annotation file downloaded from Phytozome (Zmays_181_annotation_info.txt, ftp://ftp.jgi-psf.org/pub/compgen/phytozome/v9.0/Zmays/annotation/) and significances were determined based on the Pearson chi-square test with P-values < 0.05. For each GO term, at least five genes were mapped. The significant terms were displayed in the WEGO output figure.

Maize transcription factor (TF) information was downloaded from the Plant Transcription Factor Database (PlantTFDB) v 3.0 (http://planttfdb.cbi.pku.edu.cn/), which contains 3,316 TFs corresponding to 2,231 loci classified into 55 families. We used this information to identify the TFs of the coexpressed genes.

Elongation factor 1α genes and ribosomal protein genes were obtained using the keywords ‘elongation factor Tu’ and ‘ribosomal’, respectively, to search the maize annotation file.

The annotation of zein genes were obtained from Chen [39].

### Identification of enriched cis-motifs

The genes which are among the most highly connected within a module are referred to as hub genes [23]. The top 30 hub genes in LBPGs modules were identified by ranking the intramodular connectivity of each gene. The cis-motifs in the promoter regions of hub genes within the module that contain TFs were analyzed using AME software [44]. The sequence 1-kb upstream transcriptional start site was defined as the promoter region. The promoter sequences of module genes were used as the input sequences and those of all of the maize genes (RefGen_v2 5b) were used as the control. The JASPAR CORE 2014 database was used [45]. Other parameters of AME were set at the default values. To calculate the number of genes that contain the enriched motif in corresponding modules, we downloaded the motif sequences of myb.Ph3, MEF2A, and HAT5 from the MEME suite, and searched the 1-kb upstream sequences of module genes using a custom Perl script. Because the motif sequence of Hac1 could not be downloaded from the MEME suite, its conserved sequence [ATCG][AC]CACGT[ATCG], was used.

### Analysis of the lysine contents of proteins encoded by LBPGS coexpressed genes

The lysine content of each protein was defined as the ratio of the number of lysine residues to the total number of amino acids. Protein lysine contents were calculated, and a statistical analysis was performed in R [46].

### Identification of eQTLs

Gene expression and single nucleotide polymorphism (SNP) data of 368 maize lines, which were downloaded from Maizego (http://www.maizego.org/index.html), were used for a genome-wide association study (GWAS). We used PLINK (v_1.07) [47], GCTA (v_1.24) [48], and Efficient Mixed-Model Association eXpedited (EMMAX) [49] for kinship and principal component analyses. The GWAS analysis was performed using the Genomic Association and Prediction Integrated Tool (GAPIT). For each LBPG, we used the method described by Fu et al. (2013) to identify eQTLs and possible regulators.

### Quantitative RT-PCR verification

Total RNA was isolated from B73 maize seed at 2, 6, 8, 10, 16, 20, 24 and 32 days after pollination (DAP) using an RNAprep Pure Plant kit (Tiangen, China). After digestion with DNase I, 4μg of total RNA was reverse transcribed by M-MLV reverse transcriptase (Promega, USA) with oligo (dT)_18_ universal primer. Quantitative RT-PCR (qRT-PCR) was performed using UltraSYBR Mixture (Roche, USA) on a qTOWER 2.2 real-time PCR detection system (Analytikjena, Germany). The PCR conditions consisted of 95°C for 10 min, followed by 40 cycles of 95°C for 15 s, 60°C for 1 min. PCR amplifications were performed in quadruplicate for each sample. The 2^-ΔΔCT^ method (Livak and Schmittgen, 2001) was used to calculate relative gene expression levels, which were normalized to the expression level of the maize housekeeping gene actin. All primer sequences used for qRT-PCRs are listed in S1 Table.

## Results

### Identification and phylogenetic analysis of LBPGs

To identify LBPGs in maize, the annotation of the maize lysine biosynthesis pathway on KEGG pathways was used, and a BLASTP algorithm-based search was performed in Phytozome 9.0 using the maize genome [17,50]. Using the Phytozome functional annotations, we identified 15 LBPGs: three genes encode AK (EC: 2.7.2.4), one gene encodes an aspartate-semialdehyde dehydrogenase (ASD, EC: 1.2.1.11), two genes encode DHDPS (EC: 4.3.3.7), two genes encode DapB (EC: 1.17.1.8), three genes encode LL-DAP-AT (EC: 2.6.1.83), two genes encode DapF (EC: 5.1.1.7), and two genes encode LysA (EC: 4.1.1.20) (Table 1). These enzymes are all found in the diaminopimelate variant pathway identified in Arabidopsis [10]. The homolog genes of DapD, DapC, DapE and Ddh which participate in other diaminopimelate variant pathways were not found, indicating that the pathway of Lysine biosynthesis is conserved between *Zea mays* and Arabidopsis.

**Table 1:** Lysine biosynthesis pathway enzyme genes in maize.

Gene name	Enzyme^a^	EC Num.^a^	Gene ID^b^	Module
AK1	aspartate kinase	2.7.2.4	GRMZM2G052935	blue
AK2			GRMZM2G024686	cyan
AK3			GRMZM2G104546	purple
ASD	aspartate-semialdehyde dehydrogenase	1.2.1.11	GRMZM2G076885	darkred
DHDPS1	dihydrodipicolinate synthase	4.3.3.7	GRMZM2G556131	blue
DHDPS2			GRMZM2G027835	darkred
DapB1	dihydrodipicolinate reductase	1.17.1.8	GRMZM2G104575	cyan
DapB2			GRMZM2G090241	steelblue
LL-DAP1-AT	LL-diaminopimelate aminotransferase	2.6.1.83	GRMZM2G010328	brown
LL-DAP2-AT			GRMZM2G415117	—
LL-DAP3-AT			GRMZM2G119150	—
DapF1	diaminopimelate epimerase	5.1.1.7	GRMZM2G130332	darkred
DapF2			AC182617.3_FG001	pink
LysA1	diaminopimelate decarboxylase	4.1.1.20	GRMZM2G020446	darkturquoise
LysA2			GRMZM2G090722	blue

^a^ Enzyme and EC Num. from KEGG database.

^b^ The identity of maize gene ID according to the filtered-gene set (release 5b) of reference B73 genome (AGPv2).

**Fig 1:**
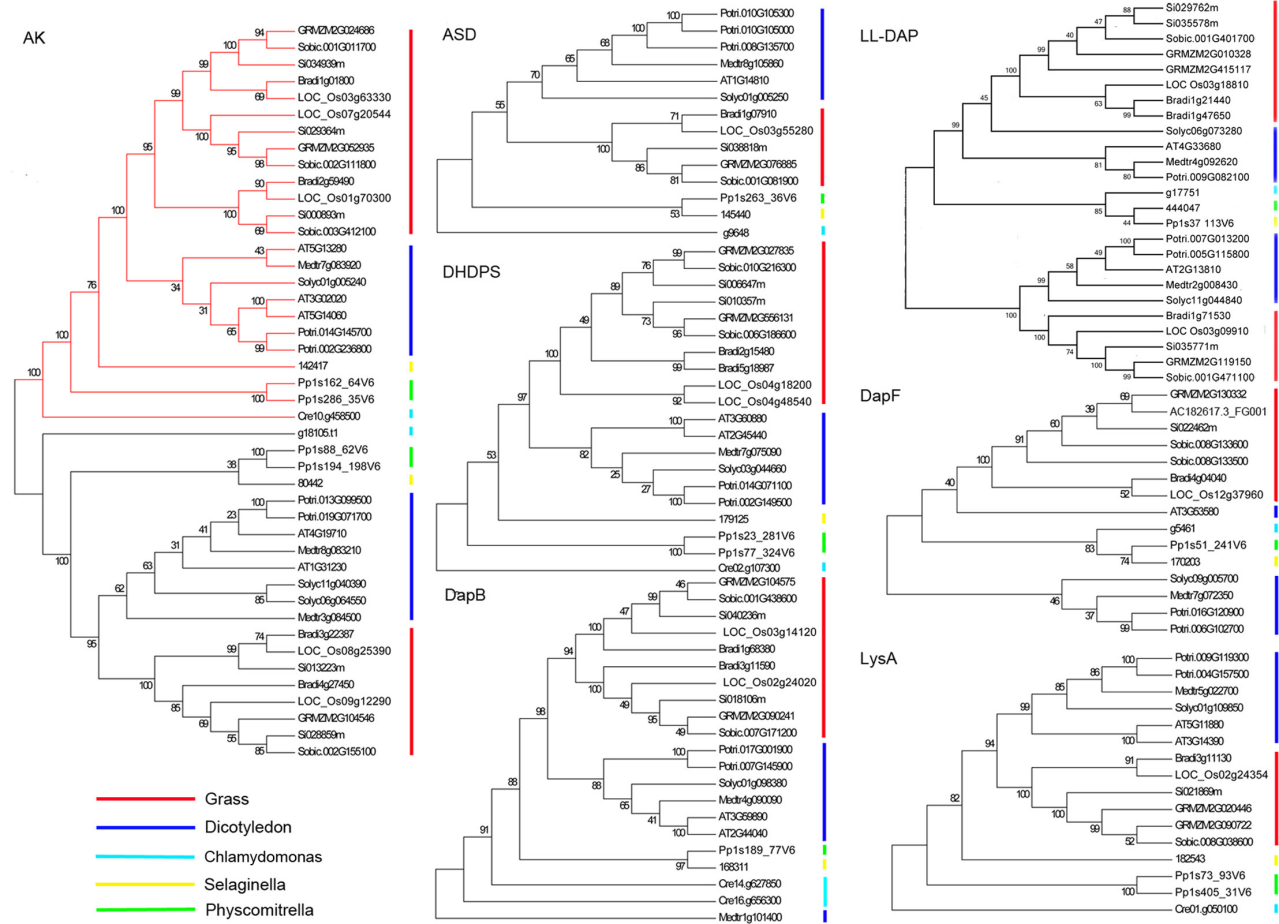
Phylogenetic trees of lysine biosynthesis pathway gene orthologs. Phylogenetic trees based on AK (aspartate kinase), ASD (aspartate-semialdehyde dehydrogenase), DHDPS (dihydrodipicolinate synthase), DapB (dihydrodipicolinate reductase), LL-DAP-AT (LL-diaminopimelate aminotransferase), DapF (diaminopimelate epimerase), and LysA (diaminopimelate decarboxylase) are shown. The AK tree contains two enzymes: the monofunctional enzyme AK (EC: 2.7.2.4; red branch) and the bifunctional enzyme HSDH (EC: 1.1.1.3; black branch)

To analyze the phylogenetic relationships of LBPGs, we used the same method described above to obtain LBPGs from four other grass plants (*Sorghum bicolor*, *Setaria italica*, *Oryza*. *sativa*, *Brachypodium distachyon*), four dicotyledon plants (*Arabidopsis thaliana*, *Medicago truncatula*, *Populus trichocarpa*, *Solanum lycopersicum*), one *Selaginellaceae* plant (*Selaginella moellendorffii*), one *Funariaceae* plant (*Physcomitrella patens*) and one *Chlamydomonadaceae* plant (*Chlamydomonas reinhardtii*) (S2 Table). MEGA5 [38] was then used to construct a neighbor-joining tree (with 500 bootstrap replicates) for each of the seven lysine biosynthesis enzymes (Fig 1). Except for gene the Medtr1g101400 in the DapB tree and gene AT3G53580 in the DapF tree, each family’s genes formed a monophyletic group. The AK tree was divided into two groups, one group contained AK1 and AK2, and the other contained AK3 (Fig 1 AK). AK3 is a homoserine dehydrogenase (HSDH, EC: 1.1.1.3), which also catalyzes the third step in the aspartate pathway as a bifunctional enzyme. In accordance with grass evolutionary relationships [51,52], the DHDPS genes of *Z*. *mays*, *S*. *bicolor* and *S*. *italica*, which are C4 grasses, were separated into two groups, while the DHDPS genes of *O*. *sativa* and *B*. *distachyon*, which are C3 grasses, were not separated(Fig 1 DHDPS). The LL-DAP genes were divided into two clades. LL-DAP1-AT, LL-DAP2-AT, and At4g33680/AtLL-DAP1-AT, and LL-DAP3-AT and At2g13810/ALD1 belong to the two clades, respectively. The AtLL-DAP-AT identified by Hudson et al. was involved in the Arabidopsis lysine biosynthesis pathway [10]. ALD1 has strong activity when lysine was used as an amino donor, indicating that it is unlikely to have the activity of LL-DAP-AT [53]. These results indicated that LL-DAP1-AT and LL-DAP2-AT may have primary functions in the lysine biosynthesis pathway in maize.

### Construction of a LBPGs coexpression network

To obtain a coexpression network of LBPGs during maize seed development, we analyzed publicly available RNA-Seq data from 21 developmental stages of B73 maize seed [39], with RPKM values representing gene expression levels. The resulting dendrogram (S1A Fig), derived from a clustered analysis of the 21 samples using 21,679 genes that were either expressed during at least two stages with RPKM ≥ 2 or at only one stage with RPKM ≥ 5, was identical to that published by Chen [39]. We used the 21,679 genes for further analyses. Because of its low expression across the 21 seed developmental stages, the LL-DAP2-AT gene was removed prior to the analysis. WGCNA [23] was then applied to construct a weighted gene coexpression network. To calculate the adjacency of the data, we chose the soft threshold power β = 16, which is the lowest power at which the scale-free topology fit index reaches 0.68 (S1B Fig).

**Fig 2:**
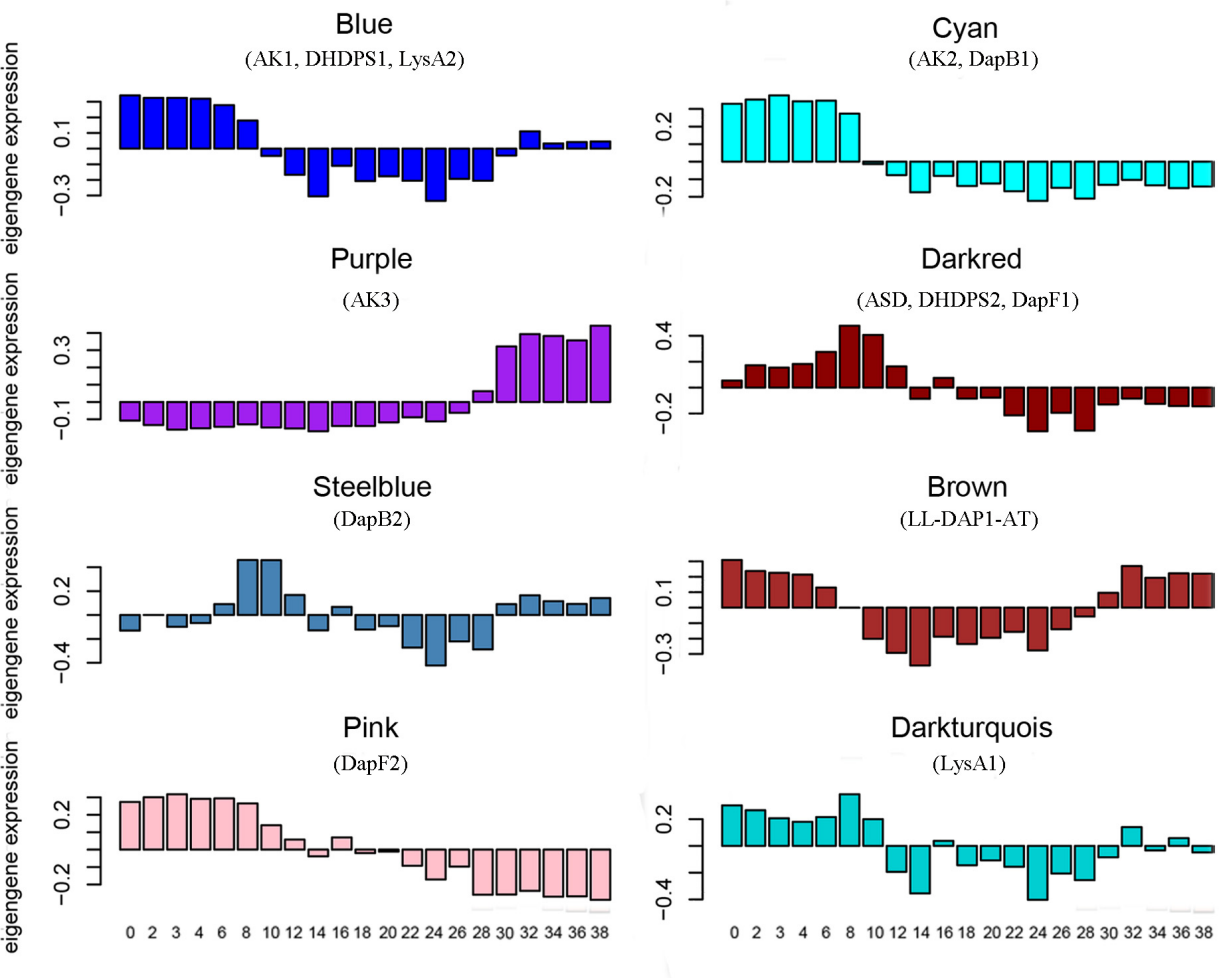
Expression pattern of LBPGs coexpressed genes. The module eigengenes are used to represent the genes expression pattern within each module. Each bar represents one sample (days after pollination) and the color represents the module color, the LBPGs was list below the module name. The value of the module eigengenes for each sample is displayed on the y axis.

The coexpression network construction yielded 24 modules (S2 Fig). The modules ranged in size from 123 genes in the skyblue module to 4,248 genes in the blue module, with 607 genes not being included in any coexpression module (grey module) (S3 Table). The LBPGs were distributed in eight modules. AK1, DHDPS1 and LysA2 were in the blue module, AK2 and DapB1 were in the cyan module, AK3 was in the purple module, ASD, DHDPS2 and DapF1 were in the darkred module, DapB2 was in the steelblue module, LL-DAP1 was in the brown module, DapF2 was in the pink module, and LysA1 was in the darkturquoise module (Table 1). There were 11 elongation factor 1α genes (S3A Fig and S4 Table) and 313 ribosomal protein genes (S3B Fig and S5 Table) coexpressed with the LBPGs. These results are in accordance with the previous reports that lysine metabolism is correlated with ribosomal proteins and elongation factor 1α genes [54,55]. The LL-DAP3-AT was in the grey module, so we removed it from the following analysis. The gene expression levels in the modules with LBPGs were further analyzed, and the results showed that the genes in the different modules had some similar features during seed development, even though these modules had different expression patterns. The expression levels of genes in the blue and brown modules were higher at the early and late stages, but lower at the middle stage. The expression level of the steelblue module was higher at 6–12 DAP and the late stage. The levels of the cyan, darkred, pink and darkturquois modules were higher only at early stage, while that of the purple module was higher only at the late stage (Fig 2).

To better understand the biological functions of the coexpressed genes, the online tool WEGO [43] was used for the GO enrichment analysis (S4 Fig). We found that several ‘Cellular Component’ GO categories, such as cell part, organelle part and protein complex, were overrepresented among the coexpressed genes. Several ‘Molecular Function’ categories, including enzyme activator, ligase, protein binding, structural constituent of ribosome, and RNA polymerase II TF, were enriched in the coexpressed genes. Cell communication and terms related to protein transport, such as localization, establishment of localization, and microtubule-based process, were the subcategories of the ‘Biological Process’ category that were enriched in the coexpressed genes. This result indicates that LBPGS coexpressed genes may play important roles in cell metabolism, specifically in protein metabolism.

### Identification and analysis of LBPG coexpressed TF genes

TF genes identify in the network modules are often important as they may regulate the expression of module genes. Using the Plant Transcription Factor Database annotation of maize TFs, we found 53 TF families containing 643 TFs coexpressed with LBPGs (S6 Table), and some of the TF families, such as MYB, bHLH, bZIP, C3H, NAC, and WRKY, contained more than 30 genes. Some TFs were hub genes in the modules. The single myb histone5 (smh5; GRMZM2G163291) was the hub gene in the brown module; homeobox-transcription factor 33 (hb33; GRMZM2G011588) and hb102 (GRMZM2G139963) in the cyan module; *Zea* AGAMOUS homolog 1 (zag1; GRMZM2G052890), MADS-transcription factor 4 (mads4; GRMZM2G032339), mads31 (GRMZM2G071620), mads6 (GRMZM2G159397), mads67 (GRMZM2G147716), mads8 (GRMZM2G102161), and mads14 (GRMZM2G099522) in the pink module; AP2-EREBP-transcription factor 206 (ereb206; GRMZM2G366434), ereb133 (GRMZM2G100727), and bZIP-transcription factor 63 (bzip63; GRMZM2G011119) in the purple module.

**Fig 3:**
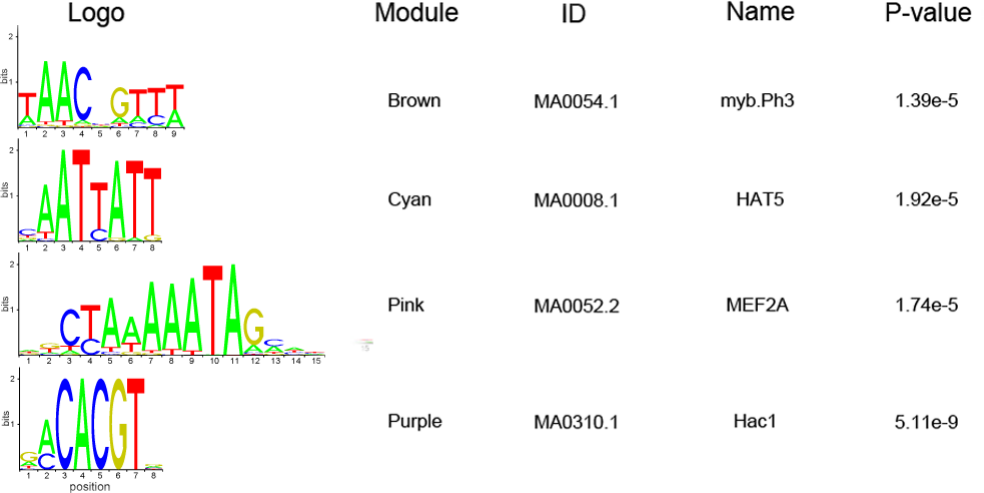
The motifs enriched in the LBPGs modules. The motifs enriched in 1-kb upstream sequences of the transcription start site of the LBPGs coexpressed module genes. (P-value was calculated by Ranksum test).

Further, TF binding sites (TFBS) were analyzed using the AME software suite (Fig 3). We found that the binding sites of myb.Ph3 (a Petunia hybrid MYB protein) were enriched in the brown module, the binding sites of HAT5 (an Arabidopsis homeobox-leucine zipper protein) in the cyan module, the binding sites of MEF2A (a human MADS box transcription enhancer factor) in the pink module, and the binding sites of Hac1 (a yeast bZIP protein) in the purple module. Additionally, the sequences of these binding sites were used to screen the gene promoter regions. The results are shown in S6 Table. Among the 3,769 genes in the brown module, there were 1,065 genes with the myb.Ph3 binding site in their promoter region. There were 703 genes containing HAT5 among the 2,250 genes in the cyan module, and there were 222 genes containing the MEF2A among the 2,139 genes in the pink module. In the purple module, there were 622 genes containing Hac1 among the 2,204 genes. These results were coincident with that the corresponding TFs were the Hub TFs in the modules, indicating that the MYB, homeobox, MADS box and bZIP families may have broad regulatory roles during maize seed development.

Remarkably, the Opaque2 gene (*O2*) (GRMZM2G015534) encoding one of the bZIP family TFs was found in the blue module and coexpressed with DHDPS1, AK1 and LysA2 (S3 Table). The inactivation of O2 could cause lysine accumulation [56]. We noticed that, except at 12–18 DAP and at 22–26 DAP, the O2 and the DHDPS1 were almost negatively correlated (Pearson’s correlation coefficient = -0.83) during maize seed development (S5A Fig). Further, we analyzed the transcriptome data of 15 DAP endosperm of the *o2* mutant published recently [57], and found that the expression level of DHDPS1 was also lower than in the wild type (S5B Fig). In addition, Li et al. analyzed the transcriptome data and chromatin immunoprecipitation data of the *o2* mutant, finally identified 35 down-regulated differentially expressed genes having O2 binding sites in their promoter regions [57]. We found that 28 of the 35 genes were in the Filtered Gene Set (5b, RefGen_v2), and that 11 of the 28 genes were in the blue module, coexpressed with O2, including *b-32* (GRMZM2G063536), *CyPPDK1* (GRMZM2G306345) and the zein genes (50-kDa, 22-kDa, 19-kDa and 14-kDa) which were the well-studied downstream genes regulated by Opaque2 (S7 Table).

### Functional annotation of DHDPS coexpressed genes

**Fig 4:**
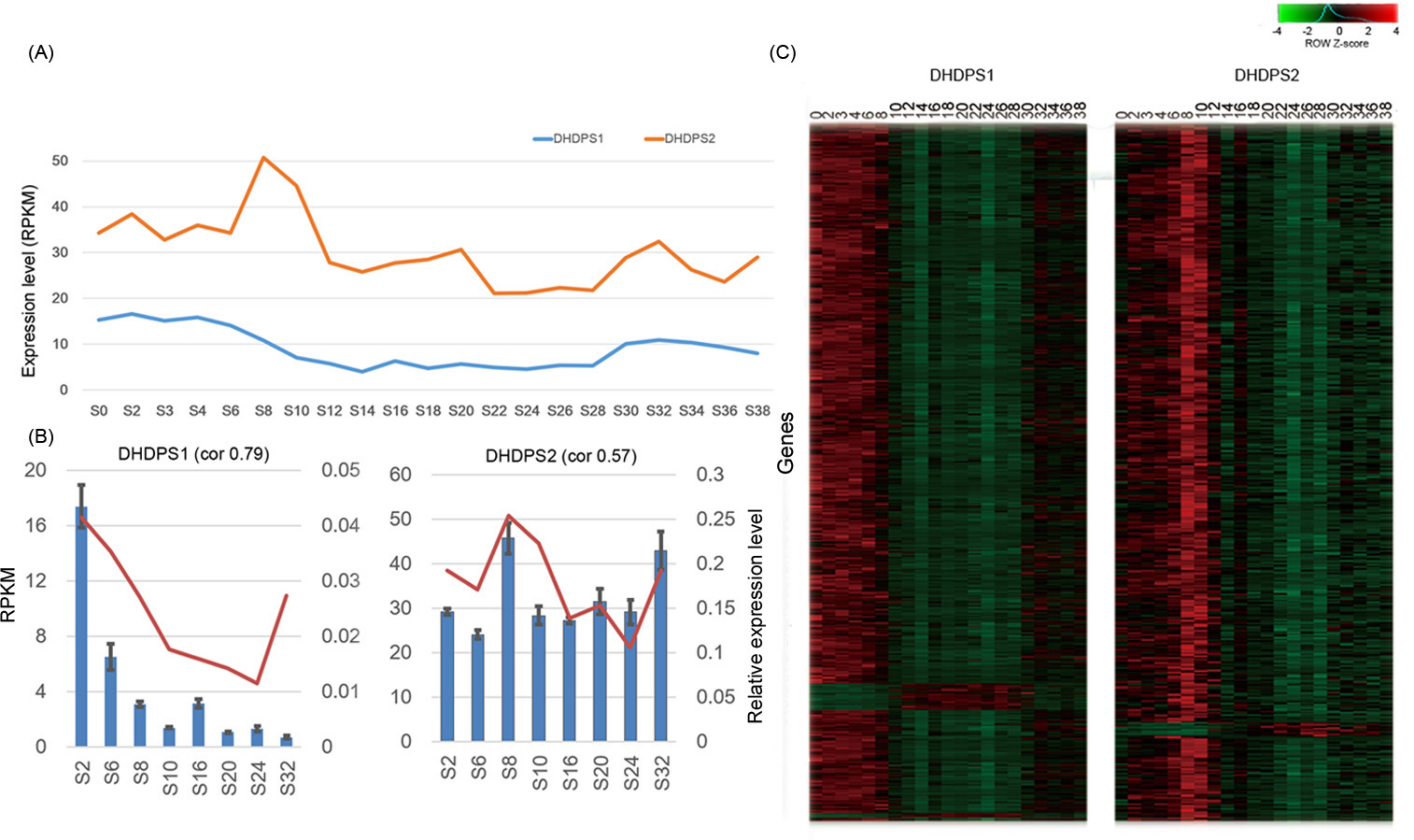
Expression patterns of dihydrodipicolinate synthase (DHDPS) genes. (A) Expression levels reads per kilobase per million reads (RPKM) of DHDPS genes in 21 maize developmental stages. (B) Quantitative real-time PCR (qRT-PCR) validation of RNA-Seq-based DHDPS expression levels. qRT-PCR expression profiles (blue bars) of DHDPS genes closely match the RNA-Seq data (red lines). The correlation value (cor) was calculated using Pearson’s correlation coefficient. The error bars in the figure represent the standard deviation. (C) Heat map of expression profiles of DHDPS-coexpressed genes (Y axes). Numbers next to the heat map represent the number of days after pollination. (The error bars represent standard deviation).

The two DHDPS genes were split into two modules in the coexpression network, and the expression level of DHDPS2 was higher than that of DHDPS1 during maize seed development (Fig 4A). To confirm the expression pattern of DHDPS genes at the maize seed developmental stages, we sampled maize seeds at 2, 6, 8, 10, 16, 20, 24, and 32 DAP for qRT-PCR validation (Fig 4B). The qRT-PCR result showed similar expression trends to those obtained by RNA-Seq. We further analyzed the expression patterns of genes that coexpressed with these two DHDPS genes (Fig 4C). We found that the DHDPS1 coexpressed genes were highly expressed at early (0–8 DAP) and late (30–38 DAP) stages, whereas DHDPS2 coexpressed genes were highly expressed at 8–10 DAP, which corresponded to the end of the early stage and the beginning of the middle stage of maize seed development.

**Fig 5:**
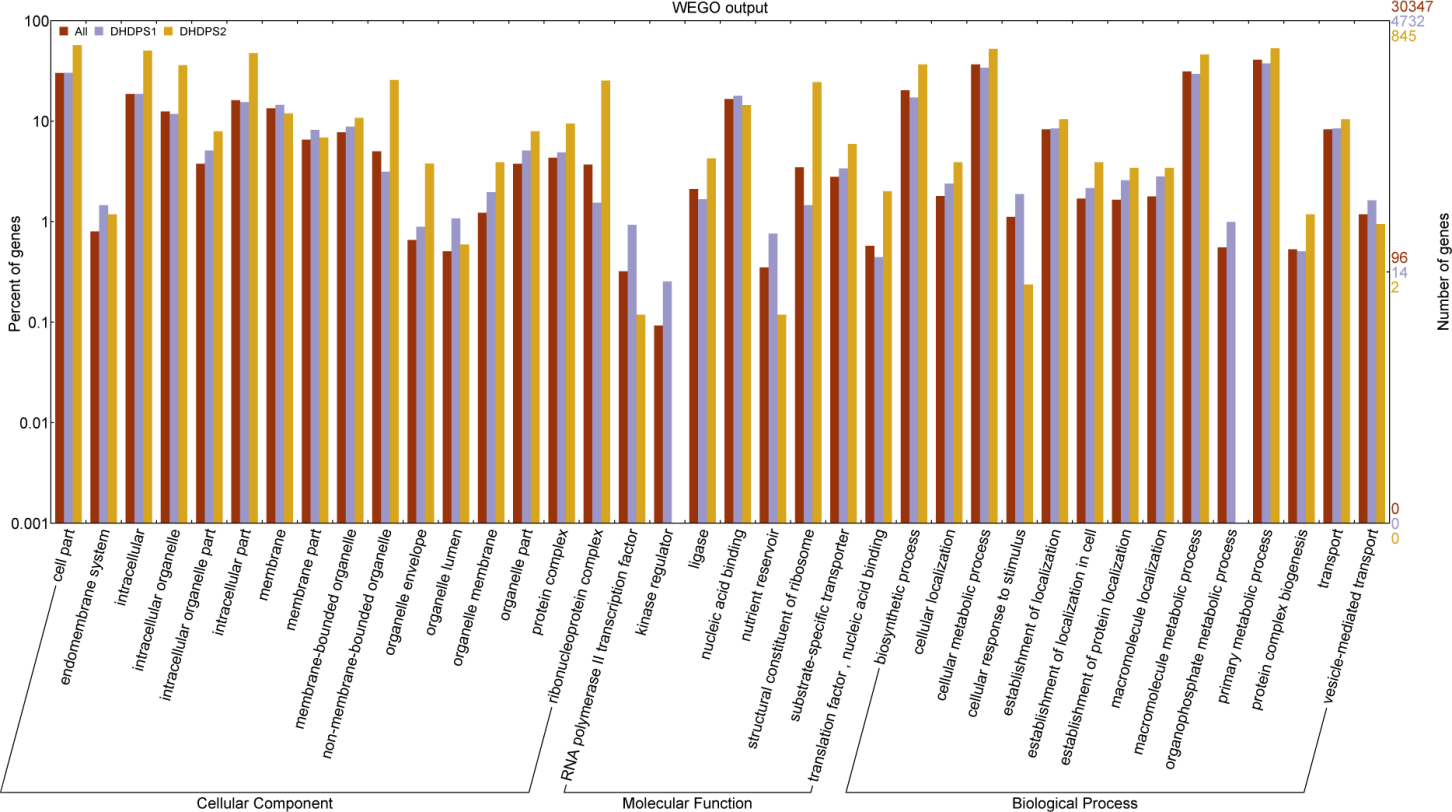
Gene Ontology (GO) functional enrichment analysis of DHDPS coexpressed genes. All represent all genes set (RefGen_v2 5b). DHDPS1 and DHDPS2 represent DHDPS1 and DHDPS2 coexpressed genes. GO categories among cell component, molecular function and biological process that show significant (*P* < 0.05) enrichment than all genes set.

To further understand the differences between the two DHDPS genes, the DHDPS coexpressed genes were analyzed using WEGO (Fig 5). The result showed that the organelle lumen, RNA polymerase II TF, nutrient reservoir and organophosphate metabolic process were enriched in DHDPS1 coexpressed genes. Twenty-two genes were related to organelle lumen, such as *abp1* (GRMZM2G116204) and *abp4* (GRMZM2G064371), which are associated with endoplasmic reticulum lumen. Sixteen genes were related to RNA polymerase II TF. Thirty genes were related to nutrient reservoir, including one 14-kDa zein, seventeen 19-kDa zeins, eleven 22-kDa zeins and one 50-kD γ-zein. Twenty-one genes in the organophosphate metabolic process GO category corresponded to the phospholipid biosynthetic process and the GPI anchor biosynthetic process. While, cell part, intracellular organelle, structural constituent of ribosome and biosynthetic process were enriched in DHDPS2 coexpressed genes. There were 93 ribosomal genes related to the structural constituent of the ribosome term. In addition, 152 genes were involved in translation processes: translational initiation process, e.g., *EIF2* (GRMZM2G030646), *SUI1* (GRMZM2G113414) and *EIF3K* (GRMZM2G115182); translational elongation process, e.g., *EF1B* (GRMZM2G029559); tRNA aminoacylation for protein translation process: *lysyl-tRNA* (GRMZM2G146589 and GRMZM2G386714). Further, we found that two GroES-like family protein genes (GRMZM2G013652 and GRMZM2G035063), which were chaperones assisting protein folding, were coexpressed with DHDPS2.

### Lysine content of proteins encoded by the coexpressed genes

**Fig 6:**
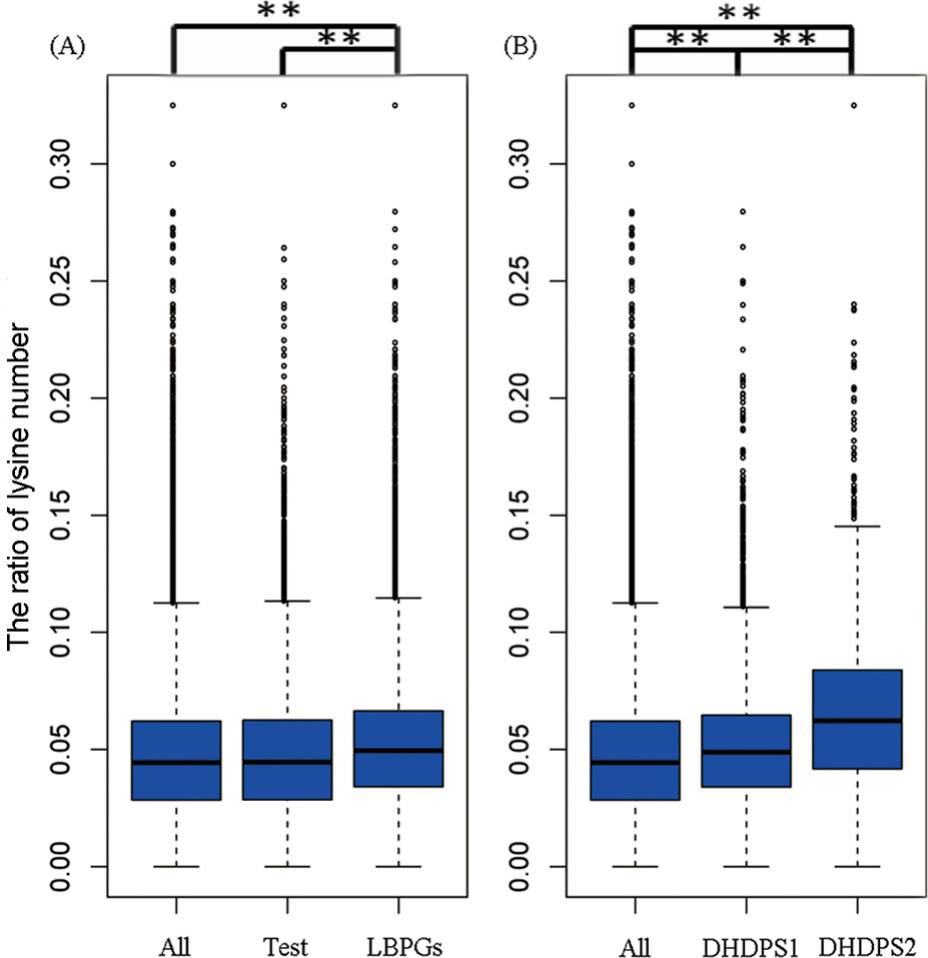
Comparison of lysine content. (A) Comparison of lysine biosynthesis pathway genes coexpressed genes (LBPGs) with the same number of randomly selected all genes set (Test) and with all genes set (All). (B) Comparison of DHDPS1-coexpressed genes (DHDPS1) and DHDPS2-coexpressed genes (DHDPS2) with all genes set (All). (** *P* < 0.01)

To analyze the differences in the lysine contents among proteins encoded by coexpressed genes, non-coexpressed genes and different modules, we defined the ratio of the number of lysines to the length of proteins as the lysine content. We found that the mean lysine content of proteins encoded by coexpressed genes (5.274%) was significantly higher (*P* < 2.200^−16^, Mann–Whitney–Wilcoxon single tail test) than that of proteins encoded by all of the gene sets (4.761%) and proteins encoded by the same number of randomly selected genes from all of the gene sets (4.790%) (Fig 6A). Next, we calculated the mean lysine content of proteins encoded by DHDPS coexpressed genes (Fig 6B). The mean lysine content of proteins encoded by DHDPS2 coexpressed genes (6.775%) was significantly higher (*P* < 2.200^−16^, Mann–Whitney–Wilcoxon single tail test) than that of proteins encoded by DHDPS1 coexpressed genes (5.129%) and all of the gene sets (4.761%). The mean lysine content of proteins encoded by DHDPS1 coexpressed genes (5.129%) was also significantly higher (*P* < 2.200^−16^, Mann-Whitney-Wilcoxon single tail Test) than that of proteins encoded by all of the gene sets (4.761%). Because the transcriptional level is not always coupled to the translational level of a given gene, and the total lysine content is also determined by the protein abundance, we analyzed the protein abundance data that was published by Walley et al. [58]. We found that the average abundance of proteins encoded by LBPG coexpressed genes was higher than that of all of the gene sets (S6A Fig). The average abundance of protein encoded by DHDPS2 coexpressed genes was higher than those encoded by DHDPS1 in the select tissues (S6B Fig). Taken together, these results suggest that the free lysine might be preferentially involved in the synthesis of high lysine content proteins encoded by LBPG coexpressed genes with higher lysine contents.

### GWAS-based discovery of LBPG eQTLs

For the GWAS analysis, we used the expression data of 28,850 maize genes and more than 1.06 million SNPs, generated from immature kernels (15 DAP) of 368 maize inbred lines [33]. GAPIT [59] was used for the association analysis of the expression levels of the 13 LBPGs. The GWAS analysis uncovered six LBPGs, DHDPS1, DapB1, DapF1, DapF2, LysA1 and LysA2, with 140 significantly associated SNPs (*P* < 1.8× 10^−6^ and a false discovery rate < 0.05). Manhattan and quantile–quantile plots for the six LBPGs resulting from the GWAS analysis are shown in S7 Fig. From the significantly associated SNPs, we identified 16 candidate eQTLs by grouping three or more SNPs when the distance between two consecutive SNPs was less than 5 kb. Because DapF1 had only one significantly associated SNP, this gene was excluded. The most significantly associated SNP in each eQTL region was defined as the lead SNP, and used to represent the eQTL. As defined by Fu et al.[33], the eQTLs with lead SNPs located more than 20-kb from their associated genes were regarded as distant eQTLs, while, the remaining eQTLs were considered to be local eQTLs. For the 16 loci, all of the possible regulators of target genes within the eQTL regions are listed in Table 2. DHDPS1 was predicted to be regulated by one local eQTL and one distant eQTL, DapB1 by two local eQTLs and four distant eQTLs, and DapF2 and LysA1 each by only one local eQTL. LysA2 was predicted to be regulated by six distant eQTLs (Table 2). Remarkably, the local eQTLs of four LBPGs (DHDPS1, DapB1, DapF2, and LysA1) all contained those target genes, indicating that the expression of these LBPGs may be regulated directly by the variation of local sequences. However, LysA2 was predicted only to be regulated by distant eQTLs. Five genes were predicted in the eQTL region, among which, *gfa1* (GRMZM2G005849) which encodes a glucosamine—fructose-6-phosphate aminotransferase existed in the LysA2 coexpression network, suggesting that GFA1 may be the main regulator of lysA2 expression.

**Table 2:** Regulation of LBPGs for maize seed Lysine biosynthesis.

Gene name	Gene ID	eQTL region	Genes included in eQTL	Lead SNP	P-value	Mode of regulation
DHDPS1	GRMZM2G556131	chr2.S_16122343- chr2.S_16128476	GRMZM2G106881	chr2.S_16125924	2.17E-11	Local
			GRMZM2G106921			
			GRMZM2G556131			
		chr2.S_16219830- chr2.S_16221283	GRMZM2G003752	chr2.S_16219830	2.17E-11	Distant
DapB1	GRMZM2G104575	chr3.S_208060073- chr3.S_208066033	GRMZM2G054470	chr3.S_208060073	1.54E-06	Distant
		chr3.S_208089798- chr3.S_208089830	GRMZM2G178227	chr3.S_208089798	1.54E-06	Distant
		chr9.S_141981972-chr9.S_141986896	GRMZM2G550865	chr9.S_141986896	9.63E-08	Distant
		chr9.S_142950294- chr9.S_142950435	GRMZM2G104575	chr9.S_142950400	1.33E-20	Local
		chr9.S_142957102- chr9.S_142960045	NA	chr9.S_142957102	5.70E-21	Local
		chr9.S_142997263- chr9.S_142997274	GRMZM2G448330	chr9.S_142997273	2.89E-08	Distant
DapF2	AC182617.3_FG001	chr3.S_127297831- chr3.S_127310088	AC182617.3_FG001	chr3.S_127300484	6.76E-10	Local
			GRMZM2G047626			
LysA1	GRMZM2G020446	chr10.S_5718864- chr10.S_5728074	GRMZM2G020446	chr10.S_5728073	6.75E-08	Local
LysA2	GRMZM2G090722	chr3.S_737330- chr3.S_741558	GRMZM5G825902	chr3.S_737330	2.55E-18	Distant
		chr3.S_230006051- chr3.S_230006131	GRMZM2G005849	chr3.S_230006128	9.08E-07	Distant
		chr3.S_231109102- chr3.S_231115552	GRMZM2G092008	chr3.S_231109102	9.04E-19	Distant
		chr3.S_231272787- chr3.S_231275101	GRMZM2G109785	chr3.S_231274978	1.74E-17	Distant
		chr3.S_231475396- chr3.S_231475415	GRMZM2G313320	chr3.S_231475396	2.51E-12	Distant
		chr7.S_141527822- chr7.S_141527824	NA	chr7.S_141527822	2.11E-11	Distant

eQTL, expression quantitative trait loci; SNP, single-nucleotide polymorphism. The eQTL region was defined by two flanking associated SNPs.

## Discussion

Because lysine is one of the most limiting essential amino acids, the improvement of the lysine content of cereal grains, especially maize, is very important. Many studies have reported that the free lysine content was elevated by inactivating the sensitivity of lysine biosynthesis enzymes (AK and DHDPS) to lysine feedback inhibition [13–15,60]. In this study, we used RNA-Seq data from 21 developmental stages of B73 maize seed to investigate the network associated with lysine biosynthesis during maize seed development.

In the coexpression network, the 13 LBPGs were split into eight modules. This result is in accordance with the generally low degree of coexpression for genes encoding enzymes involved in metabolic pathways [61,62]. Further, the eight modules tended to have higher expression levels at the early stage and lower expression levels at the middle stage of maize seed development. One explanation is that at the early stage of maize seed development, more free lysine needs to be synthesized to meet the increasing demands of protein synthesis, while at the middle stage the amount of free lysine transport from the peduncle vascular sap or other tissues to the developing seed may provide enough free lysine for protein synthesis [63,64].

According to the GO enrichment analysis, the main functions of LBPG coexpressed genes were related to cellular protein activities. These associations with cellular proteins indicated that the lysine biosynthesis genes had a close relationship with protein metabolic networks. In plants, transcriptional regulation is very important in many metabolic pathways, as TFs tend to control multiple pathway steps [65]. We identified 643 TFs in the network (S6 Table), many of which were members of TF families regulating seed-related biological processes, such as seed protein metabolism and seed development. For example, the bZIP family regulates lysine metabolism [66,67]; MYB, B3 and DOF families control seed storage protein genes expression [68–71]; and C3H, AP2, bHLH, HB-other, MIKC and NAC families regulate seed development [72,73]. We also found that some TFs were hub genes within a module, such as the seven MADS-box TFs in the pink module. Further, we analyzed the TFBSs of the modules that contain TFs in the hub genes, and found the corresponding binding sites were enriched in the module (Fig 3). Additionally, we analyzed the regulatory network of LBPGs, and detected 5 local eQTLs and 11 distant eQTLs for 5 LBPGs (Table 2). DHDPS1 was predicted to be regulated by a local eQTL. The two LysAs were regulated by eQTLs, LysA1 was regulated by a local eQTL, and LysA2 was regulated by six distant eQTLs. These results will improve our understanding of lysine biosynthesis regulation. Together, the results indicated that genetic control is involved in the lysine biosynthesis, and that the lysine biosynthesis is regulated at the transcriptional level.

In *Escherichia coli*, the lack of GroE causes the level of dihydrodipicolinate synthase to be significantly reduced [74]. In our network, we found two GroES-like family protein genes were coexpressed with DHDPS2. DHDPS2 may require chaperones to fold, and its activity is regulated by molecular chaperones in maize, as well as in *Escherichia coli*.

DHDPS is the core enzyme in the diaminopimelate pathway, and its structure has been solved in several plants recently, such as Arabidopsis [75] and grape [76]. DHDPS activity is influenced by the changes in the kinetic parameters and is feedback-inhibited by lysine. Meanwhile, in Arabidopsis, it had been shown that transcriptional regulation of the DHDPS gene exerts a primary control on lysine synthesis [77]. In this work, we studied the DHDPS genes’ coexpression network in maize seed. We identified two genes encoding DHDPS in maize seed. They share 73.45% identity at the nucleotide level. However, in the coexpression network, the two genes were separated into different modules and there were three different points in the coexpression network.

First, the two DHDPS genes had different expression levels. The expression level of DHDPS2 was higher than that of DHDPS1 during seed development (Fig 4A). The same result is also found in the seedlings of Arabidopsis in which the DHDPS2 expression level is higher than that of DHDPS1 [78]. Mutant analyses of Arabidopsis showed that *dhdps2* has an increase in threonine, but *dhdps1* does not [78]. These indicated that the DHDPS2 controls the flux in lysine biosynthesis. When the activity of DHDPS2 decreased, aspartate semialdehyde, which is the common material for the synthesis of lysine, threonine and methionine, is used to synthesize more threonine [79].

Second, according to the GO enrichment analysis, the DHDPS1 coexpressed genes were enriched in the GO term of nutrient reservoir activity, RNA polymerase II TF and organophosphate metabolic process, while the DHDPS2 coexpressed genes were enriched in cell part, organelle, ribosomal, and biosynthesis process (Fig 5).

Third, the lysine content of proteins encoded by genes coexpressed with DHDPS2 was significantly higher than that of proteins encoded by DHDPS1 coexpressed genes (Fig 6B). These results imply that the DHDPS1 and DHSPS2 genes may contribute differently to lysine synthesis during maize seed development.

Previous studies showed that free lysine accumulated transiently at intermediate stages of tobacco seed development [80]. Free lysine accumulation in Arabidopsis seeds is positive correlated (r = 0.7704) with DHDPS expression levels [6,81]. DHDPS2 and their coexpressed genes were highly expressed in maize seeds at 8–10 DAP (Fig 4C) and in central starchy endosperm at 8 DAP (S8 Fig). Considering that in the physiological process of seed development, the most active stage of endosperm starch and storage protein synthesis is at 12–15 DAP (Kynast, 2012)—as is the spatiotemporal expression (Fig 4C and S8 Fig) and the functions of the coexpressed genes—we think it is very likely that DHDPS2 is responsible for the synthesis of some high lysine content proteins related to translation, such as ribosomal proteins that have a high protein abundance at 8–12 DAP (S6C Fig). These high lysine content proteins are involved in and/or contribute to the synthesis of the proteins involved in the later stage of seed development.

There is another question about the role of DHDPS1 in maize seed. In Arabidopsis, the two isoforms of DHDPS (DHDPS1 and DHDPS2) contribute differently to lysine biosynthesis [78]. During the seed development stage, we found that the DHDPS1 had a close relationship with O2 (Pearson’s correlation coefficient = −0.83). Varisi et al. reported that there was no clear evidence that *o1*, *o2*, *fl1* and *fl2* genes influenced DHDPS activity in developing maize seeds [82]. This discrepancy may be because the expression level of DHDPS2 was higher than that of DHDPS1 in maize seed, and the reduced activity of DHDPS1 had no effect on the total DHDPS activity level. DHDPS1 and DHDPS2 might make different contributions to lysine biosynthesis in maize seed as well as in Arabidopsis. Based on the MaizeGDB gene expression profile, we found that DHDPS1 was expressed highly in thirteenth leaves and in leaves after pollination (http://www.maizegdb.org/gene_center/gene/GRMZM2G556131), while DHDPS2 was expressed highly in seed (http://www.maizegdb.org/gene_center/gene/GRMZM2G027835). This suggested that DHDPS1 and DHDPS2 may have specific functions in different tissues. DHDPS1 and DHDPS2 may have important roles in leaves and seeds, respectively.

Recently, Walley et al. reported that there was a poor correlation between mRNA levels and protein abundance in maize seed endosperm at 12 DAP (r = 0.414) and embryo at 20 DAP (r = 0.413) [58]. Here, we compared the DHDPS2 coexpressed gene proteome data with the RNA-Seq data of maize seed endosperm at 8, 10 and 12 DAP, and the correlation coefficients were 0.34, 0.39 and 0.43, respectively. The results were consistent with those of Walley et al.[58]. Interestingly, the mRNA levels of LBPGs showed a relatively high correlation (r = 0.85) to protein abundances in maize endosperm at 12 DAP in Walley et al.’s study [58]. Ponnala et al. also reported that, in maize leaves, the photosynthetic genes show the highest correlation when leaves serve as the carbon source [83]. These results indicated that the correlations between mRNA and protein abundance have a wide range depending on the gene function and growth stage.

In summary, we constructed a complex LBPGs coexpression network based on RNA-Seq data from 21 different maize seed developmental stages. After determining that DHDPS genes exhibit different expression levels and expression patterns, we comparatively analyzed DHDPS1 and DHDPS2 coexpressed genes and determined that DHDPS1 and DHDPS2contribute differently to lysine biosynthesis in maize seed. We also analyzed the function of LBPG coexpressed genes and identified some potential regulators of lysine biosynthesis, such as certain TF families, and local and distant eQTLs. Although further biochemical and molecular experiments are needed for verification, our results provide a foundation for future studies on the lysine biosynthesis pathway network in maize seed.

## Accession Numbers

The sources of data underlying the findings described in our manuscript can be found in the S9 Table.

## Supporting Information

S1 Fig Clustering dendrogram of the 21 maize seed developmental stages and the network topology for various soft-thresholding powers. (A) Clustering dendrogram of the 21 maize seed developmental stages based on their Euclidean distance by WGCNA. (B) The left panel shows the scale-free fit index (y-axis) as a function of the soft-thresholding power (x-axis). The right panel displays the mean connectivity (degree, y-axis) as a function of the soft-thresholding power (x-axis). The S represent the day after pollination of seed.
(TIF)

S2 Fig Expression pattern of 24 module genes. The module eigengenes are used to represent the genes expression pattern within each module. Each bar represents one sample (0, 2, 3, 4, 6, 8, 10, 12, 14, 16, 18, 20, 22, 24, 26, 28, 30, 32, 34, 36, 38, days after pollination) and the color represents the module color. The value of the module eigengenes for each sample is displayed on the y axis.
(TIF)

S3 Fig Coexpression relationship of lysine biosynthesis pathway gene (LBPGs) with elongation factors 1α genes and ribosomal protein genes. (A) Coexpression relationship of LBPGs with elongation factors 1α genes. (B) Coexpression relationship of LBPGs with ribosomal protein genes. The red node are the LBPGs, the green node are the elongation factors 1α genes. The blue node are the ribosomal protein genes.
(TIF)

S4 Fig Gene Ontology (GO) functional enrichment analysis of LBPG coexpressed genes. All represent all genes set. LBPGs represent LBPGs coexpressed genes. GO categories among cell component, molecular function and biological process that show significant (*P* < 0.05) enrichment than all working set genes.
(TIF)

S5 Fig The expression pattern of DHDPS1 and Opaque2. (A) The expression level of DHDPS1 and Opaque2 during maize seed development. (The S represent the seed, the number represent the days after pollination) (B) The expression level of DHDPS1 and Opaque2 in 15 DAP endosperm of wide type and *o2* mutant. (FPKM: fragments per kilobase of exon per million fragments mapped).
(TIF)

S6 Fig The average protein abundance encoded by coexpressed genes. (A) The protein abundance that encoded by lysine biosynthesis pathway genes coexpressed genes (LBPGs) and all genes set (All). (B) The protein abundance that encoded by DHDPS1 coexpressed genes (DHDPS1) and DHDPS2 coexpressed genes (DHDPS2). (C) The protein abundance of ribosomal protein that encoded by DHDPS2 coexpressed ribosomal genes (the number represent the days after pollination).
(TIF)

S7 Fig Manhattan and quantile-quantile plots from the genome-wide association analysis. In the Manhattan plot shown on the left, the dashed horizontal line corresponds to the Benjamin-Hochberg-adjusted significance threshold (*P* < 1.8 × 10^−6^). The quantile-quantile plot is shown on the right.
(TIF)

S8 Fig Heat map of expression profiles of DHDPS-coexpressed genes. (Y axes is the DHDPS-coexpressed genes) in the ten compartments of 8 DAP maize seed. Abbreviations: AL, aleurone; BETL, basal endosperm transfer layer; CSE, central starchy endosperm; CZ, conducting zone; EMB, embryo; ESR, embryo-surrounding region; NU, nucellus; PC: placento-chalazal region; PE, pericarp; PED, vascular region of the pedicel.
(TIF)

S1 Table Table for Primer sequences used for quantitative RT-PCR validation of RNA-Seq. (XLSX)

S2 Table Table for genes of lysine biosynthesis pathway enzymes of difference species. (XLSX)

S3 Table Table for the summary of the modules and coexpression gene number of LBPGs. (XLSX)

S4 Table Table for Coexpression relationship of Lysine biosynthesis pathway genes with elongation factors 1α genes. (XLSX)

S5 Table Table for Coexpression relationship of Lysine biosynthesis pathway genes with ribosomal protein genes. (XLSX)

S6 Table Table for LBPGs coexpression transcription factor. (XLSX)

S7 Table Table for down regulated differentially expressed (DEGs) genes (which were DHDPS1 coexpressed genes) with O2 Binding Site in their promoter region. (XLSX)

S8 Table Table for The DEGs coexpressed with DHDPS. (XLSX)

S9 Table Table for The source of Data underlying the findings described in our manuscript. (XLSX)
