# Supplementary figures and images for: Genome-Wide Analysis of the Lysine Biosynthesis Pathway Network during Maize Seed Development
Source: PLoS One. 2016 Feb 1;11(2):e0148287. doi: 10.1371/journal.pone.0148287 (PMC4734768; doi:10.1371/journal.pone.0148287)

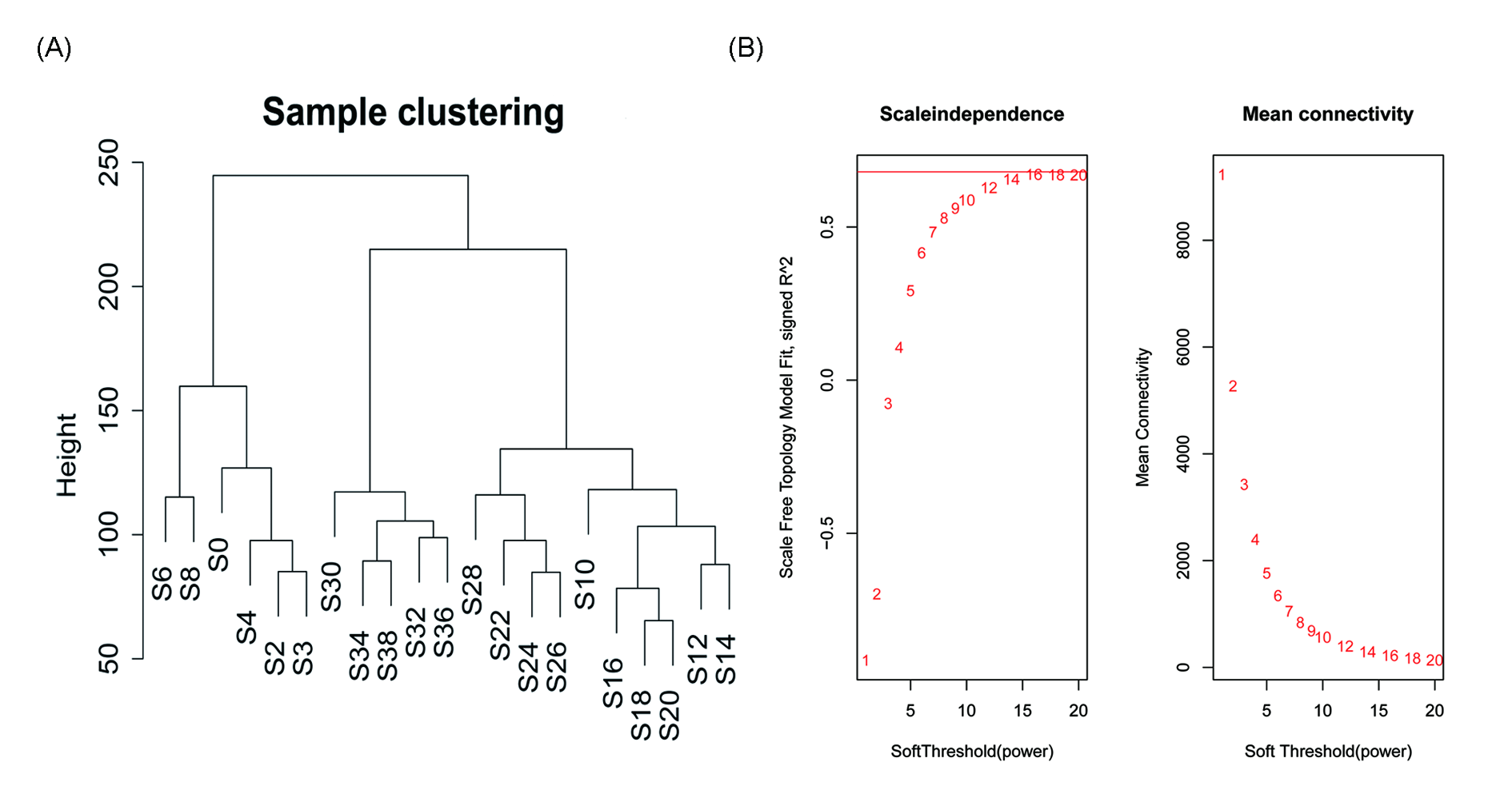

Supplement: S1 Fig — (A) Clustering dendrogram of the 21 maize seed developmental stages based on their Euclidean distance by WGCNA. (B) The left panel shows the scale-free fit index (y-axis) as a function of the soft-thresholding power (x-axis). The right panel displays the mean connectivity (degree, y-axis) as a function of the soft-thresholding power (x-axis). The S represent the day after pollination of seed. (TIF) [file pone.0148287.s001.tif]

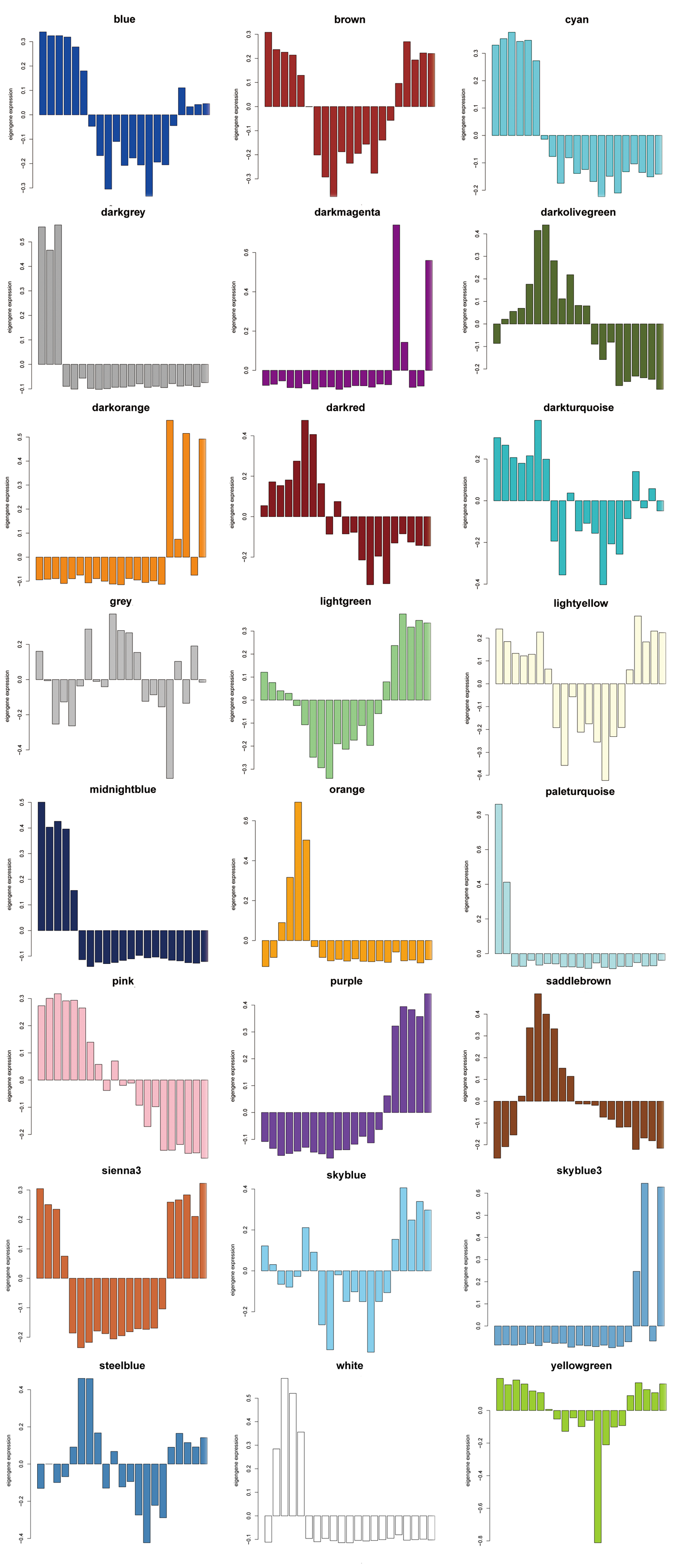

Supplement: S2 Fig — The module eigengenes are used to represent the genes expression pattern within each module. Each bar represents one sample (0, 2, 3, 4, 6, 8, 10, 12, 14, 16, 18, 20, 22, 24, 26, 28, 30, 32, 34, 36, 38, days after pollination) and the color represents the module color. The value of the module eigengenes for each sample is displayed on the y axis. (TIF) [file pone.0148287.s002.tif]

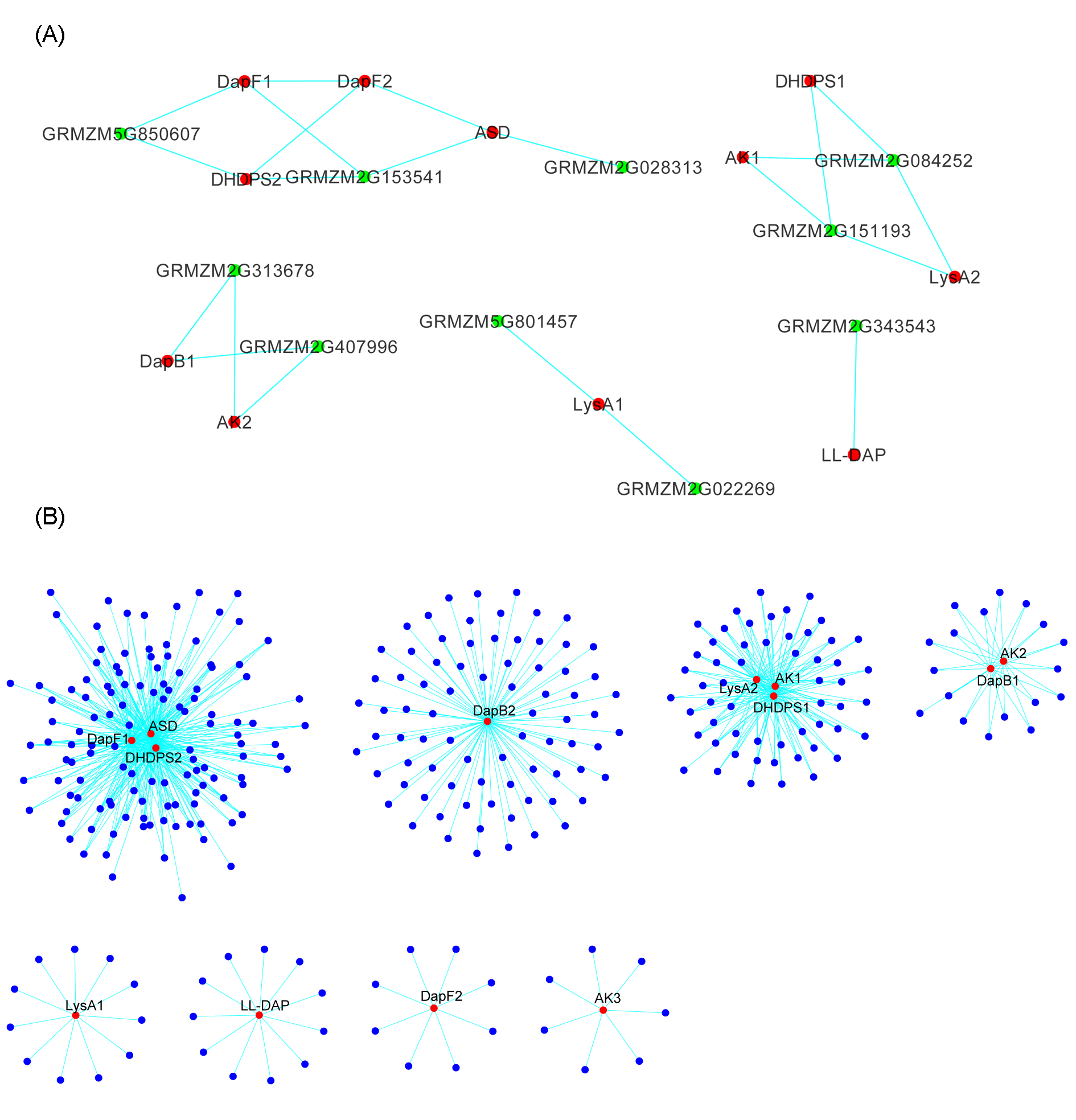

Supplement: S3 Fig — (A) Coexpression relationship of LBPGs with elongation factors 1α genes. (B) Coexpression relationship of LBPGs with ribosomal protein genes. The red node are the LBPGs, the green node are the elongation factors 1α genes. The blue node are the ribosomal protein genes. (TIF) [file pone.0148287.s003.tif]

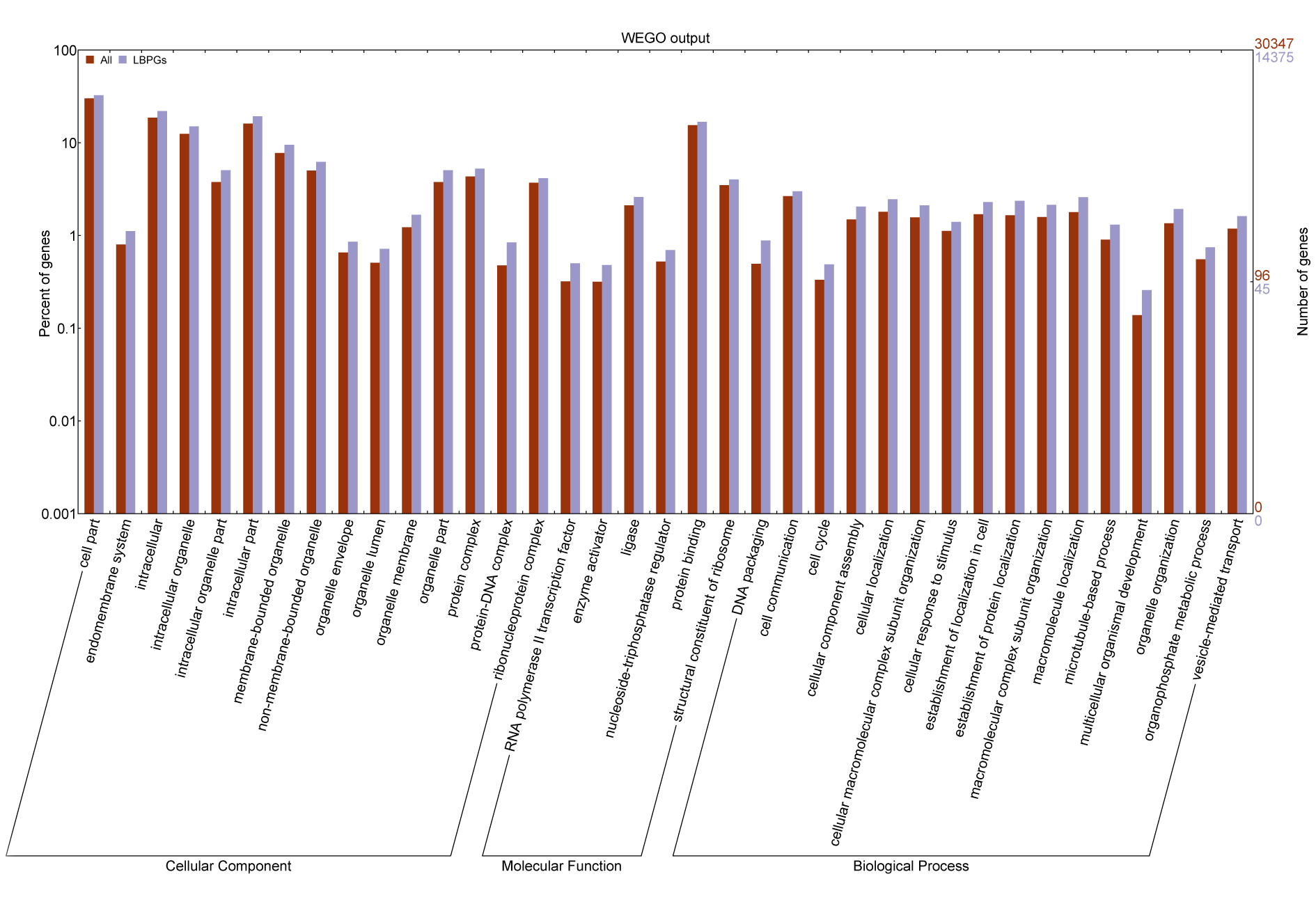

Supplement: S4 Fig — All represent all genes set. LBPGs represent LBPGs coexpressed genes. GO categories among cell component, molecular function and biological process that show significant (P < 0.05) enrichment than all working set genes. (TIF) [file pone.0148287.s004.tif]

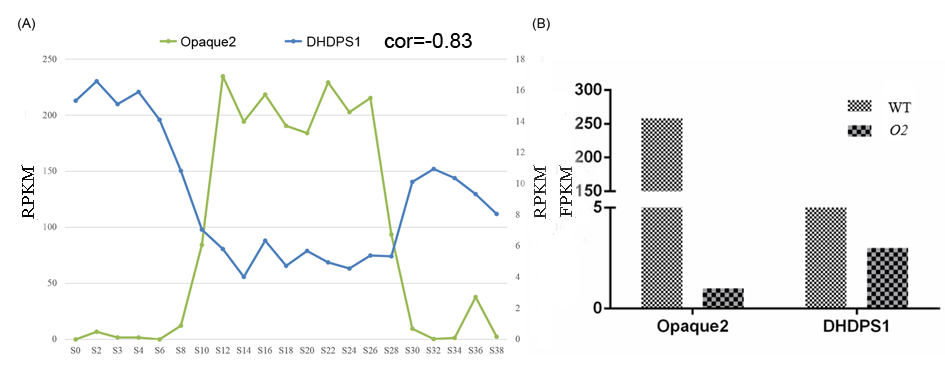

Supplement: S5 Fig — (A) The expression level of DHDPS1 and Opaque2 during maize seed development. (The S represent the seed, the number represent the days after pollination) (B) The expression level of DHDPS1 and Opaque2 in 15 DAP endosperm of wide type and o2 mutant. (FPKM: fragments per kilobase of exon per million fragments mapped). (TIF) [file pone.0148287.s005.tif]

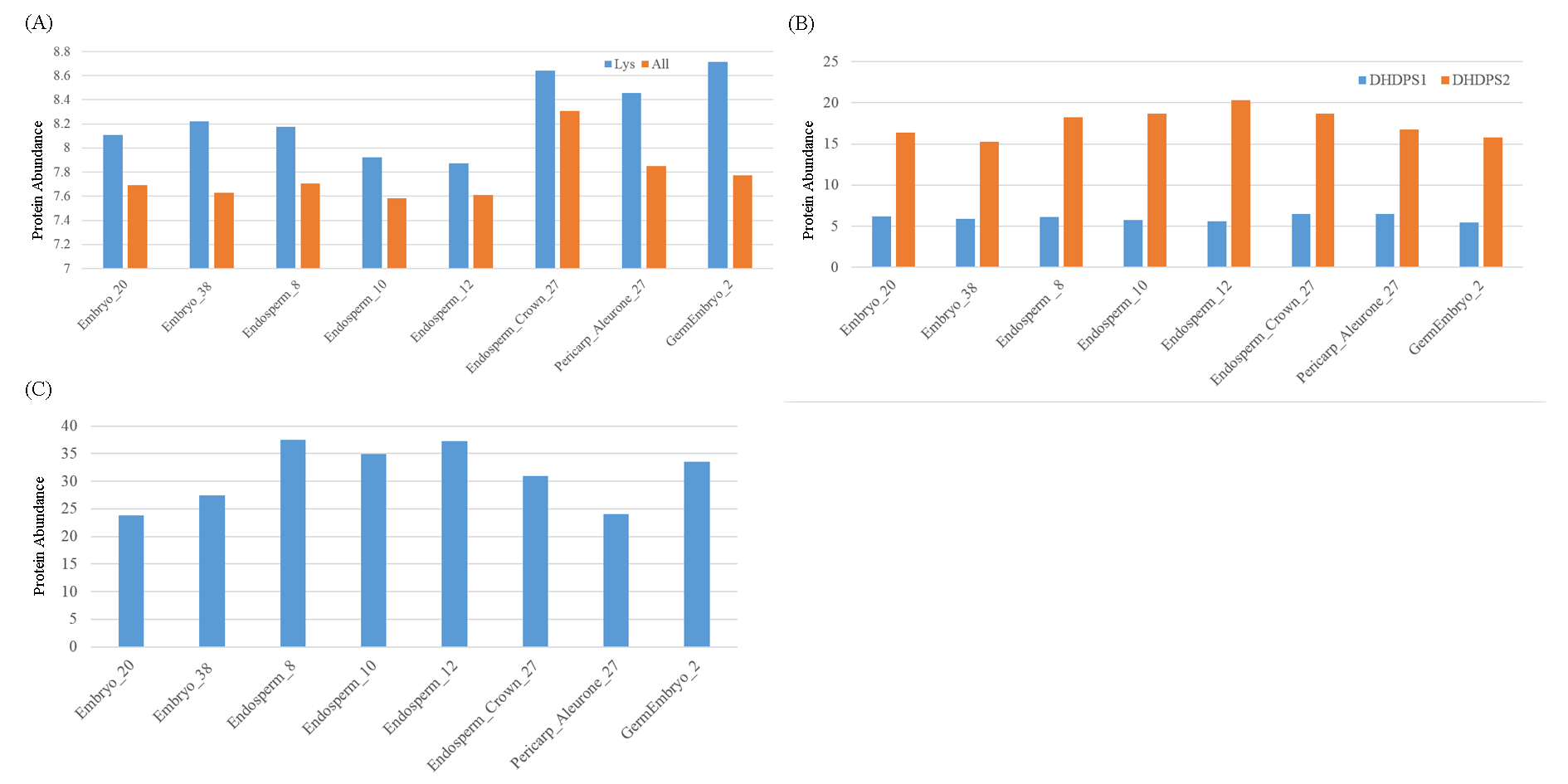

Supplement: S6 Fig — (A) The protein abundance that encoded by lysine biosynthesis pathway genes coexpressed genes (LBPGs) and all genes set (All). (B) The protein abundance that encoded by DHDPS1 coexpressed genes (DHDPS1) and DHDPS2 coexpressed genes (DHDPS2). (C) The protein abundance of ribosomal protein that encoded by DHDPS2 coexpressed ribosomal genes (the number represent the days after pollination). (TIF) [file pone.0148287.s006.tif]

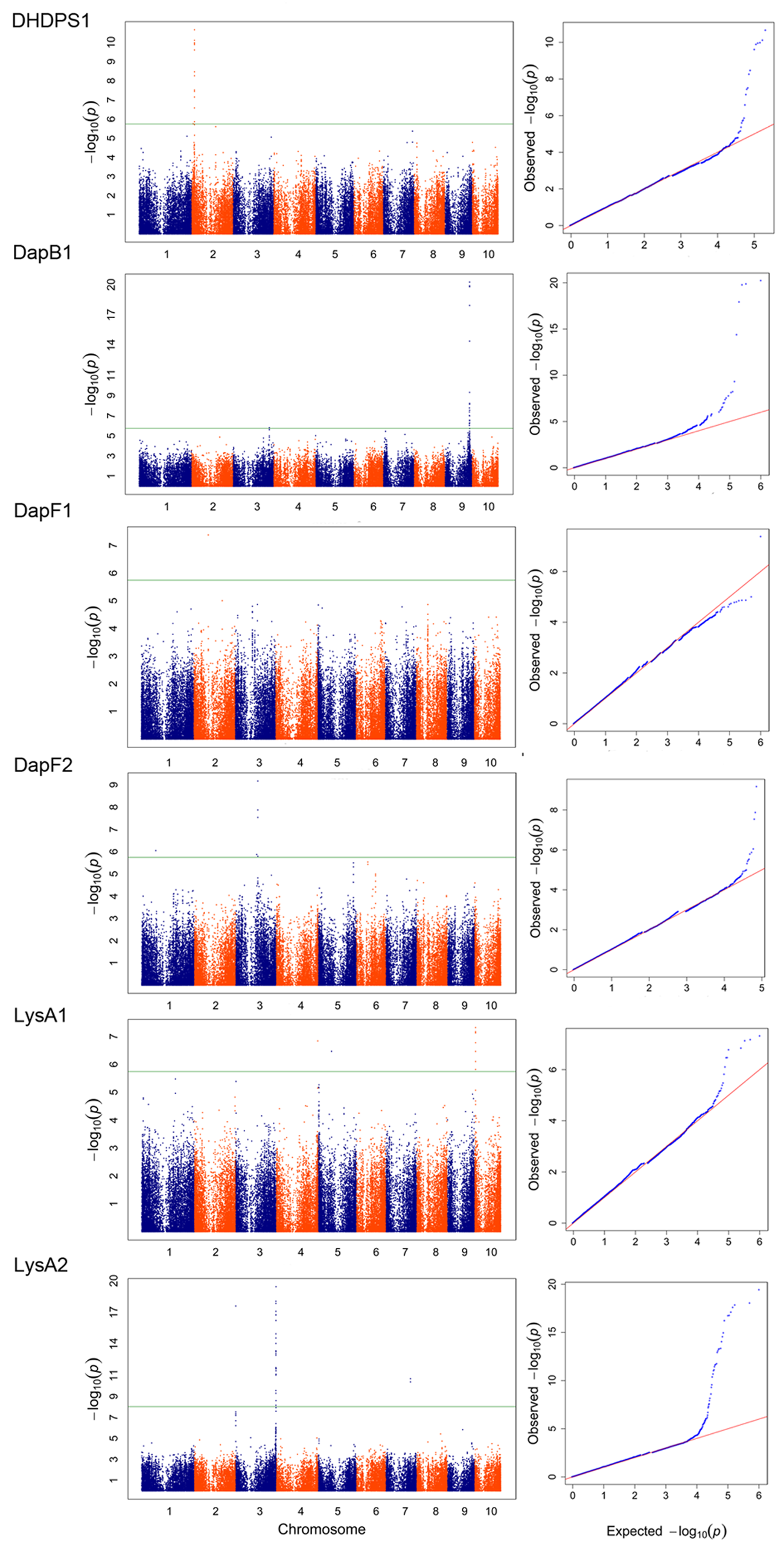

Supplement: S7 Fig — In the Manhattan plot shown on the left, the dashed horizontal line corresponds to the Benjamin-Hochberg-adjusted significance threshold (P < 1.8 × 10^−6^). The quantile-quantile plot is shown on the right. (TIF) [file pone.0148287.s007.tif]

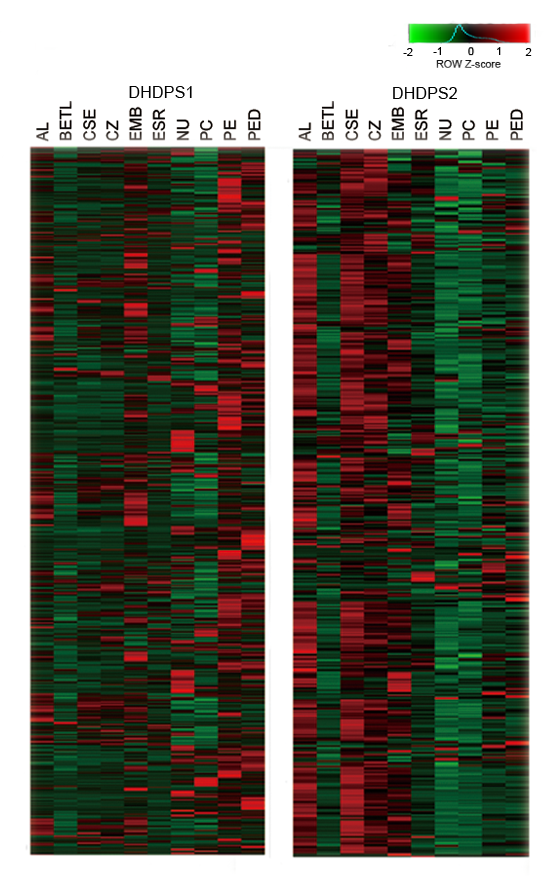

Supplement: S8 Fig — (Y axes is the DHDPS-coexpressed genes) in the ten compartments of 8 DAP maize seed. Abbreviations: AL, aleurone; BETL, basal endosperm transfer layer; CSE, central starchy endosperm; CZ, conducting zone; EMB, embryo; ESR, embryo-surrounding region; NU, nucellus; PC: placento-chalazal region; PE, pericarp; PED, vascular region of the pedicel. (TIF) [file pone.0148287.s008.tif]
